# Axially‐confined *in vivo* single‐cell labeling by primed conversion using blue and red lasers with conventional confocal microscopes

**DOI:** 10.1111/dgd.12412

**Published:** 2017-12-13

**Authors:** Atsushi Taniguchi, Yukiko Kimura, Ikue Mori, Shigenori Nonaka, Shin‐ichi Higashijima

**Affiliations:** ^1^ National Institutes of Natural Sciences National Institute for Basic Biology Okazaki 444‐8585 Japan; ^2^ National Institutes of Natural Sciences Okazaki Institute for Integrative Bioscience National Institute for Basic Biology Okazaki 444‐8787 Japan; ^3^ Neuroscience Institute of the Graduate School of Science Nagoya University Nagoya 464‐8602 Japan

**Keywords:** axial resolution, confocal laser‐scanning microscope, Dendra2, primed conversion, zebrafish

## Abstract

Green‐to‐red photoconvertible fluorescent proteins have been found to undergo efficient photoconversion by a new method termed primed conversion that uses dual wave‐length illumination with blue and red/near‐infrared light. By modifying a confocal laser‐scanning microscope (CLSM) such that two laser beams only meet at the focal plane, confined photoconversion at the axial dimension has been achieved. The necessity of this custom modification to the CLSM, however, has precluded the wide‐spread use of this method. Here, we investigated whether spatially‐restricted primed conversion could be achieved with CLSM without any hardware modification. We found that the primed conversion of Dendra2 using a conventional CLSM with two visible lasers (473 nm and 635 nm) and a high NA objective lens (NA, 1.30) resulted in dramatic restriction of photoconversion volume: half‐width half‐maximum for the axial dimension was below 5 μm, which is comparable to the outcome of the original method that used the microscope modification. As a proof of this method's effectiveness, we used this technique in living zebrafish embryos and succeeded in revealing the complex anatomy of individual neurons packed between neighboring cells. Because unmodified CLSMs are widely available, this method can be widely applicable for labeling cells with single‐cell resolution.

## Graphical abstract

The previous method (A) and the current method (B) of primed conversion. Spatially‐confined primed conversion can be achieved with this simple method.

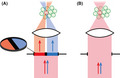

## Introduction

Genetic labeling of cells with fluorescent proteins (FPs) and subsequent optical monitoring have become popular techniques for revealing cell morphology and monitoring dynamic developmental processes, such as cell migration, morphological changes and divisions. A commonly used approach is to generate transgenic organisms in which FPs are expressed under the control of cell‐type‐ or tissue‐ specific promoters, allowing for genetic restriction of FP expression to specific types of cells (i.e., Higashijima *et al*. 2000). With this approach by itself, however, single‐cell resolution imaging has often been difficult because a large number of cells belonging to the same type express FP. For example, visualizing neurite morphology *in vivo* with single‐cell resolution is often difficult due to the high density and complexity of neural packing in the nervous system. Photoconvertible fluorescent proteins (PCFPs) can alleviate this problem. Green‐to‐red PCFPs, such as Kaede (Ando *et al*. 2002), mEOS2 (McKinney *et al*. 2009), and Dendra2 (Gurskaya *et al*. 2006), change their spectral properties upon light‐induced photoconversion. This feature allows researchers to highlight a small number of cells in a globally fluorescent cellular context. Nevertheless, perfect single‐cell labeling in tightly packed tissues such as those that make up the nervous system has been difficult even with PCFPs. The traditional method for efficient photoconversion requires the use of violet/UV single‐photon excitation (i.e., 405 nm), which is not confined to the axial dimension. Under conditions where many cells express PCFPs in a three‐dimensional manner, cells that are located above and below the target cells undergo photoconversion when exposed to a single‐photon converting beam. Two‐photon excitation with high‐power, femtosecond‐pulsed infrared lasers is a popular way to obtain excitations that are confined to the Z‐axis, but it has been reported that negligible photoconversion occurs in any of the currently available PCFPs (Dempsey *et al*. 2015), precluding the use of two‐photon microscopes for photoconversion.

**Figure 1:**
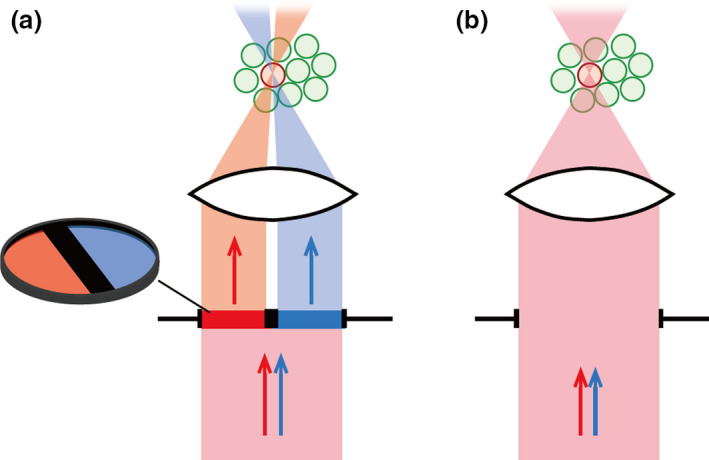
Schematics of dual laser illumination for primed conversion. (a) Schematic of the technique originally reported by Dempsey *et al*. (2015). The author implemented a specially‐designed filter plate in the excitation light path of a CLSM. The filter plate consists of two semi‐circular optical filters transmittable for blue laser or infrared laser with a thick opaque separator in the middle. With this design, the two laser beams can be aligned to meet only in a defined 3D volume, allowing for efficient axial confinement of photoconversion. (b) Schematic of the technique reported in the current study. Samples are simultaneously illuminated by blue and red laser beams in the standard manner used in CLSM. Spatially‐confined primed conversion can be achieved with this simple method.

Recently, Dempsey *et al*. (2015) reported a new approach that enables axially confined photoconversion. The authors first found an unusual phenomenon of two‐stage primed photoconversion in Dendra2 and mEOS2: a near‐infrared light (i.e., 730 nm laser) applied with the ‘priming’ blue light (i.e., 488 nm laser) simultaneously or in rapid succession was shown to lead to efficient photoconversion (Dempsey *et al*. 2015). To achieve axially‐confined photoconversion, the authors implemented a specially‐designed filter plate in the excitation light path of a confocal laser‐scanning microscope (CLSM). The filter plate, which is called a ‘confinement filter’, consists of two semi‐circular optical filters transmittable for either the priming beam (blue laser) or the converting beam (infrared laser) with a thick opaque separator in the middle. With this design, the two laser beams can be aligned to meet only in a defined 3D volume, allowing efficient axial confinement of photoconversion (see, Fig. 1a). The axial resolution (full width at half‐maximum, FWHM, at the axial dimension) was approximately 5 μm, allowing single‐cell‐resolution imaging in neuronal tissues (Dempsey *et al*. 2015).

Although the method described above was innovative, it has not become popular, presumably because the implementation of a custom‐built ‘confinement filter’ into the light path of a CLSM with a high‐power infrared laser (i.e., a two‐photon microscope) is a big hurdle for many biologists. If confined primed conversion could be achieved with CLSMs without any special customization, it would be of huge benefit. In this study, we unexpectedly found that spatially‐confined primed conversion can be achieved using a conventional CLSM with two visible blue and red lasers (Klementieva *et al*. 2016) and a high NA objective lens (NA, 1.30). The axial resolution, measured in the form of FWHM, was below 5 μm, which is comparable to that achieved by the original method with the ‘confinement filter’. As proof of this method's effectiveness, we used this technique in living zebrafish larvae and succeeded in revealing the complex anatomy of individual neurons packed between neighboring cells.

## Materials and methods

### Protein expression

The protein coding sequence of Dendra2 (Gurskaya *et al*. 2006) was cloned into the pGEX4‐T vector and transformed in BL21 cells. Two mL of a LB overnight pre‐culture was transferred to a 180 mL culture containing 1 mM IPTG. Cultures were incubated at 30°C for 16 h. Cells were centrifuged for 5 min at 5000 *g*. GST‐Dendra2 fusion proteins were extracted, and purified using MagneGST Protein Purification System (Promega) according to the manufacturer's instructions. The elution buffer used was 10 mM of Tris‐HCl (pH 8) containing 50 mM of Glutathione. Purified proteins were concentrated using Amicon Ultra‐2 mL filter (Merck). Protein concentration was estimated using a spectrophotometer (SmartSpec 2000: Bio‐Rad) at a wavelength of 260 nm.

### Polyacrylamide protein gels for in vitro analyses

120 μL Tris‐HCl solution (50 mM, pH 8) containing GST‐Dendra2 fusion proteins at a concentration of 6 mg/mL was quickly mixed with 30 μL 40% acrylamide/bis‐acrylamide (29:1 ratio, Wako), 0.8 μL 10% ammonium persulfate (Wako) and 0.15 μL TEMED (Wako). The final concentration of acrylamide was 6%. The mixture was poured into a glass‐bottom dish (35 mmΦ; hole size, 14 mmΦ), followed by a placement of a coverslip (18 mm × 18 mm) at the top. This resulted in the formation of a cross‐linked gel within 20 min. Then, 1.5% low‐melting‐point agarose solution was added to the dish and a dish cap was placed. After solidification of the agarose, the polyacrylamide gel containing GST‐Dendra2 fusion proteins was viewed by either an upright or an inverted confocal microscope (FV1200, Olympus).

### Microscopic observation of in vitro samples

**Figure 2:**
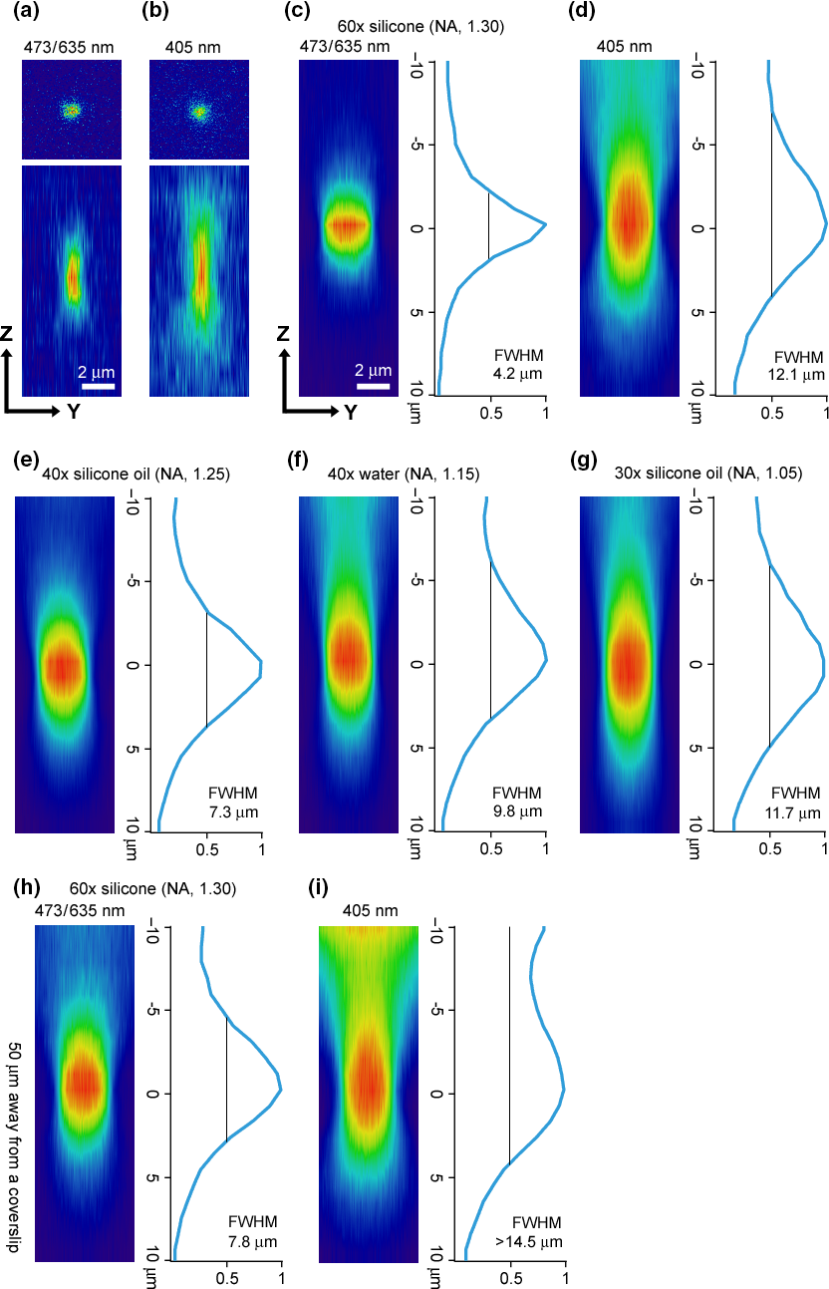
Blue/red dual‐laser illumination of Dendra2 in CLSMs leads to spatially‐confined primed conversion. In all experiments, GST‐Dendra2 fusion proteins embedded in a 6% polyacrylamid gel were photoconverted. In (a–d), a 60 × (silicone oil immersion; NA, 1.30) objective lens was used to view the region that was 20 μm away from the coverslip. In (e–g), three different objective lenses were used to view the same region. In (h) and (i), deeper regions in the Z dimension (50 μm) were viewed. (a, b) Photoconversion by spot illumination of 473/635 nm (a) and 405 nm (b). Top panels show X–Y images of red channel at the focal plane, while bottom panels show Y‐Z images. (c, d) Rectangular (31 μm × 3 μm) regions were first illuminated by 473/635 nm (c) or 405 nm (d). Then, the voxel images of red channel were projected to the X‐dimension. Y–Z sections of the projected images are shown in (c) and (d). The right panel for each figure shows the intensity profile in the Z dimension. Full‐width at half‐maximum (FWHM) was 4.2 μm for the illumination of 473/635 nm (c) and 12.1 μm for the illumination of 405 nm. (e‐g) The same experiments shown in (c) were carried out with three different objective lenses. (e) 40 × (silicone oil immersion; NA, 1.25) objective lens. FWHM was 7.3 μm. (f) 40 × (water immersion; NA, 1.15) objective lens. FWHM was 9.8 μm. (g) 30 × (silicone oil immersion; NA, 1.05) objective lens. FWHM was 11.7 μm. (h) The same experiment shown in (c) was carried out except that a deeper region (50 μm away from a coverslip) was viewed. FWHM was 7.8 μm. (i) The same experiment as in (h) was carried out except that 405 nm illumination was used. FWHM was more than 14.5 μm.

Imaging experiments in vitro were performed on Olympus FV1200 confocal microscopes. Diode‐pumped solid‐state (DPSS) laser sources were used. The maximum power for each laser line was the following: 56 mW for 405 nm, 20 mW for 473 nm, 100 mW for 559 nm, and 20 mW for 635 nm. The objective lenses used were: UPLSAPO30xS (silicone oil immersion; NA, 1.05), UAPON40xW340 (water immersion; NA1.15), UPLSAPO40xS (silicone oil immersion; NA, 1.25), and UPLSAPO60xS (silicone oil immersion; NA, 1.30). The green form of Dendra2 was imaged with the 473‐nm laser, whereas the red form was imaged with the 559‐nm laser. Imaging was performed in 512 × 512 pixel mode. Zoom factor was changed according to the magnification of the objective lenses: 13.2 for 30 ×, 10 for 40 ×, and 6.6 for 60 ×. Under these conditions, 512 pixels corresponds to approximately 31 μm. In most of the experiments, scanning speed was set as fast as possible (2.0‐μs pixel dwell). In some of the experiments, scanning speed was purposely slowed down to 0.2 ×  (10.0‐μs pixel dwell) or 0.05 ×  (40.0‐μs pixel dwell). For the single‐photon photoconversion, the 405‐nm laser was attenuated to 0.1–0.2%. For primed photoconversion, the 473‐nm laser was attenuated to 0.2 %, while the 635‐nm laser was used at maximum power (100%). The output power at the sample was around 1–2 μW for 405 nm, 2–4 μW for 473 nm, and 1–2 mW for 635 nm (the light power at the sample varied depending on the objective lens used). The duration of illumination was set such that a sufficient amount of the red form of Dendra2 appeared under observation with the 559‐nm laser; typically, this duration was on the order of seconds (1–10 s). Point illumination or rectangular illumination was performed using the ‘bleach’ mode of the FV1200. For rectangular illumination, a fixed area of 512 × 50 pixels (31 μm × 3 μm) was illuminated. To calculate the FWHM of the photoconversion along the Z‐axis, images were projected to the X axis, and average fluorescence intensity along the Z axis was measured. To generate the panels in Figure 2, we performed the photoconversion experiments in adjacent regions of the same gel.

### Fish care and strains

Zebrafish adults, embryos, and larvae were maintained at 28.5°C. Experiments were performed at room temperature (25–28°C). All procedures were performed in compliance with the guidelines approved by the animal care and use committees of the National Institutes of Natural Sciences. Animals were staged according to days post fertilization (dpf).

The Dendra2 coding sequence was cloned into a Tol2 vector, and Tg[UAS:Dendra2] was generated using the 14 ×  UAS sequence as a promoter as described previously (Kimura *et al*. 2013). Tg[HuC:Gal4‐VP16] (Kimura *et al*. 2008), Tg[chx10:Gal4] (Kimura *et al*. 2013), Tg[eng1b‐hs:Gal4] (Kimura *et al*. 2014), and Tg[vachta‐hs:Gal4] (generated in this study) were used as driver strains to express Dendra2 in distinct classes of (or all of) neurons.

### Labeling individual neurons in the zebrafish embryo with primed conversion

At 3‐4 dpf, Dendra2‐expressing larvae were anesthetized and embedded in low‐melting‐point agarose as described previously (Higashijima *et al*. 2004b). Primed conversion was achieved using the 60 × /1.30‐NA silicone‐oil objective as described above. Typically, circular ROI with a diameter around 4‐5 μm was illuminated with a 473‐nm priming beam (attenuated to 0.2%) and a 635‐nm converting beam (100%) for several seconds to a minute. In some experiments, the same neurons were illuminated twice with a half hour of an interval. The samples were left for 2–4 h before observation to allow Red Dendra2 to diffuse throughout the whole neuron including dendrites and axons. Imaging of neuronal morphology was performed using either an Olympus FV1200 CLSM (a 60 × /1.30‐NA or a 30 × /1.05‐NA silicone‐oil‐immersion objective lens) or a Leica SP8 CLSM (a HCX IRAPO L 25 × /0.95‐NA or a HC PLAPO 63 × /1.20 water‐immersion objective lens).

## Results

### Spatially confined primed conversion with a regular CLSM with blue and red lasers

Klementieva *et al*. (2016) have recently shown that a combination of blue and visible red (not infrared) laser (i.e., 633 nm) can evoke primed conversion (see also, Mohr *et al*. 2017), opening up the possibility of performing primed conversion using CLSMs without expensive high‐power infrared laser sources. Upon seeing this report, we initially aimed to establish spatially‐confined primed conversion by installing a custom‐made ‘confinement filter’ into the light path of a CLSM using blue (473 nm) and red (635 nm) lasers (Fig. 1a; Dempsey *et al*. 2015; Mohr *et al*. 2017). During the course of this study, however, we unexpectedly noticed that, even without a ‘confinement filter’, photoconversion volume appeared to be much more restricted with primed conversion in the axial dimension than it is with regular photoconversion (405 nm illumination). This led us to perform a quantitative analysis of the axial resolution that could be achieved using a conventional CSLM with blue and red lasers (Fig. 1b).

Previous studies have shown that the optimum power ratio between priming beam (blue) and converting beam (red or near‐infrared) is around the range of 1 to 100‐1000 (Dempsey *et al*. 2015; Klementieva *et al*. 2016). Therefore, we used a ratio of around 1:500 (see Methods) in the present study. For all experiments, we used the maximum power (100%) for the 635 nm laser. This was because a high‐power converting beam is necessary for efficient primed conversion (Dempsey *et al*. 2015; Klementieva *et al*. 2016) and because, even with this laser set to 100%, the power of the red visible laser (1–2 mW at the sample) was still lower than that of the infrared (titan‐sapphire) laser power used in the original report (5–10 mW at the sample; Dempsey *et al*. 2015).

Among various green‐to‐red photoconverting proteins, Dendra2 was shown to be the most efficient for primed conversion (Dempsey *et al*. 2015). Therefore, we chose to use Dendra2 in the current study. GST‐Dendra2 fusion proteins were expressed in bacteria. The proteins were then purified and embedded in 6% polyacrylamide gel. GST‐Dendra2 located in the gel 20 μm away from the coverslip was viewed through a CLSM using a 60 ×  silicone‐oil‐immersion objective lens (NA, 1.30), and photoconversion was induced by point illumination with either 405 nm or 473/635 nm. Both procedures resulted in the appearance of red Dendra2 at the focal plane (top panels of Fig. 2a,b; X–Y image of the focal plane). We then obtained serial optical sections of the red channel. Inspection of Y–Z sections revealed that photoconversion volume achieved by dual‐wavelength illumination was much more spatially confined than that achieved by 405 nm illumination in the axial (Z) dimension (bottom panels of Fig. 2a,b). To better quantify the axial resolution, a rectangular area (31 μm × 3 μm) was illuminated with 473/635 nm or 405 nm illumination. Then, voxel images of the red channel were projected to the X‐dimension. Y–Z sections of the projected images are shown in Figure 2c,d. The right panel in each figure shows the intensity profile of the red channel in the axial dimension. For the 405 nm illumination, full‐width at half‐maximum (FWHM) of red Dendra2 along the Z‐axis was more than 12 μm (Fig. 2d), consistent with that in the previous report (Dempsey *et al*. 2015). By contrast, FWHM for the 473/635 nm illumination was markedly reduced to 4.2 μm (Fig. 2c). This value is comparable with that obtained by the installation of the custom‐made ‘confinement filter’ (Fig. 1a; Dempsey *et al*. 2015), indicating that confined primed conversion can be achieved without any modification to a CLSM with blue and red lasers.

Next, we varied several parameters and examined their effects on the axial resolution. First, we tested different objective lenses: 40 × (silicone oil, NA 1.25), 40 × (water, NA 1.15), and 30 × (silicone oil, NA 1.05) objective lenses were tested in addition to the 60 × (silicone oil, NA 1.30). As shown in Figure 2c,e–g, the axial resolutions were functions of NA: in general, a bigger NA lens yielded a better result for the axial resolution. We then examined the effects of depth in the sample by examining regions located 50 μm away from the coverslip (note that the results described above were obtained at 20 μm away). For both 405 nm and 473/635 nm illuminations, FWHM became wider at greater depth (Fig. 2h,i). Yet its value remained at 7.8 μm for the 473/635 nm illumination (Fig. 2h). This value is a range of single cell resolution.

The previous study showed that the scanning speed is a factor in restricting axial dimension: Confined primed conversion was most efficient when a region of interest was irradiated using a short pixel dwell time (Dempsey *et al*. 2015). Thus, we examined whether scanning speeds affect the resolution in the Z‐axis in our method. The experiments described thus far were performed using the fastest scan speed (2.0‐μs pixel dwell). We tested the following two slower scan speeds: 0.2 × (10.0‐μs pixel dwell) and 0.05 × (40.0‐μs pixel dwell). In both cases, the resolution in the Z‐axis worsened: approximately 20% increase of FWHM in the 0.2 × scan speed and an approximately 30% increase of FWHM in the 0.05 × scan speed (data not shown). Thus, an in the original report, the fastest scanning produced the best results in our method.

### Revealing single‐neuron morphology with confined primed conversion

Visualizing detailed neurite morphology *in vivo* has been a challenging issue, due to the difficulty in distinguishing between the neurites of densely packed neighboring cells. The confined primed conversion technique described above could allow us to highlight single neurons in the nervous system with a high resolution. To verify this, we used larval zebrafish as a model system because the animals are optically transparent.

**Figure 3:**
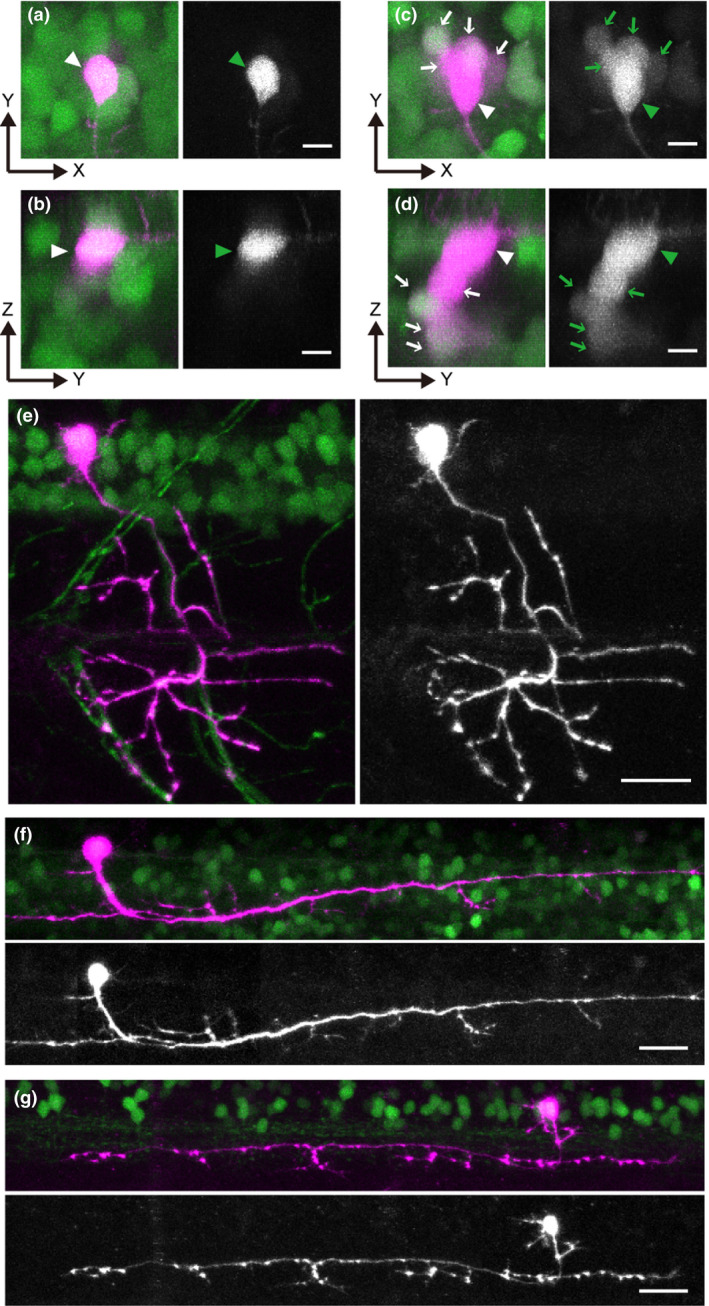
Spatially confined primed conversion enables individual neuron labeling in tightly packed neural clusters in living larval zebrafish. Photoconversion experiments were performed in the spinal cords of 3‐to‐4‐dpf larvae expressing Dendra2. (a‐d) Photoconversion of Dendra2 in Tg[HuC:Gal4]; Tg[UAS:Dendra2] with primed conversion (a and b) or conventional 405 nm illumination (c and d). (a and c) are stacked images of the X‐Y dimension, while (b and d) are stacked images of the X‐Z dimension. In each image, the left panel shows both green and red channels (red channel is shown in magenta), while the right panel shows only the red channel (shown in black‐and‐white). The primed conversion successfully highlights only the targeted cell (arrowheads in a and b). In contrast, with 405nm‐illumination, not only the targeted cell (arrowheads in c and d) but also cells located nearby (primarily those located in the Z dimension) express red Dendra2 (arrows in c and d). (e) Photoconversion of Dendra2 in Tg[vachta‐hs:Gal4]; Tg[UAS:Dendra2] with primed conversion. The morphology of an individual trunk motoneuron is clearly revealed in its entirety. (f) Photoconversion of Dendra2 in Tg[chx10:Gal4]; Tg[UAS:Dendra2] with a primed conversion. The morphology of an individual chx10‐positive neuron (a neuron whose axon descends on the same side of the spinal cord; Kimura et al., 2006) is clearly revealed. (g) Photoconversion of Dendra2 in Tg[eng1b‐hs:Gal4]; Tg[UAS:Dendra2] with primed conversion. The morphology of an individual eng1b‐positive neuron (a neuron whose axon ascends on the same side of the spinal cord; Higashijima et al., 2004a) is clearly revealed. Scale bars: 5 μm in a–d; 20 μm in e–g.

We established Tg[UAS:Dendra2] transgenic zebrafish strains. Then, one of the strains was crossed to a pan‐neuronal Tg[HuC:Gal4] driver strain (Kimura *et al*. 2008). The compound transgenic larvae expressed Dendra2 in all neurons. The spinal cord in 3 dpf animals was chosen for imaging analyses. As shown in Figures 3a–d, a large number of neurons expressed the green‐form of Dendra2 in the compound transgenic fish. Figure 3a,b are images obtained after primed conversion with 473/635 nm illumination to highlight a single neuron (Fig. 3a consists of stacks of X–Y images, while Fig. 3b consists of stacks of X–Z images). The targeted neuron (arrowhead) clearly shows the red‐form of Dendra2, while red signals in neighboring neurons are negligible. For comparison, we also performed photoconversion by illuminating samples with a 405 nm laser. As shown in Figures 3c,d, not only the targeted neuron (arrowhead) but also neurons located below it (arrows) show detectable levels of red Dendra2. This indicates that primed conversion is indeed useful for highlighting individual neurons *in vivo*.

Next, we used several other Gal4 driver lines to express Dendra2 in distinct types of neurons. The lines we used were Tg[vachta‐hs:Gal4] for cholinergic neurons (primarily, motoneurons), Tg[chx10:Gal4] for ipsilateral descending neurons (Kimura *et al*. 2013), and Tg[eng1b‐hs:Gal4] for a ipsilateral ascending neurons (Kimura *et al*. 2014). In all cases, primed photoconversion (473/635 nm illumination) successfully revealed the morphologies of neurons at a single cell resolution (Fig. 3e–g). Characteristic axonal projection patterns are clearly discernible for all types of neurons, indicating the usefulness of primed conversion for revealing neuronal morphology at a single‐cell resolution.

## Discussion

In this study, we have shown that axially‐confined primed conversion can be achieved using conventional CLSMs with blue and red visible lasers. An axial resolution of approximately 5 μm (FWHM, 4.2 μm) is possible with a 60 × objective lens (NA, 1.30). This value is comparable to that obtained in a previous report involving a custom‐made modification of a two‐photon CLSM (Dempsey *et al*. 2015). With its greater simplicity, the current method will be applicable for many types of photoconversion experiments.

Spatially‐confined primed conversion is likely to be caused by non‐linear effects. Because the reaction requires the absorption of dual photons (blue and red), it is reasonable to speculate that the reaction rate is not simply proportional to the light energy applied. Rather, some kind of non‐linear integration effects are likely to occur for the two‐step chemical reactions to be completed. This would explain why photoconversion efficiency is dramatically condensed near the focal plane in the case of primed conversion. The previous study suggested the presence of a relatively‐long‐lived (approximately 3.75 ms) intermediate state upon priming beam illumination (Dempsey *et al*. 2015). This means that two photons are not absorbed simultaneously in primed conversion. This is different from two‐photon excitation in which the absorption of two photons must occur simultaneously (Denk *et al*. 1990). In this sense, the non‐linear effects (the sharpness of the axial resolution) in primed conversion are likely to be milder than those in typical two‐photon excitation.

For primed conversion experiments, a typical energy ratio between the priming beam (blue) and the converting beam (red/infrared) is 1:500–1000 (Dempsey *et al*. 2015) (this study). Thus, the source of the converting beam (red or infrared) must have sufficiently high power to enable practical experiments. In this study, the maximum power of the red laser source was 20 mW, and we used it at its full power for all experiments; under this condition, laser power at the sample was 0.5–2.0 mW. Illumination time for photoconversion under this condition was within a practical range (several seconds for in‐vitro experiments and several seconds to a minute for in‐vivo experiments). In situations where only lower red laser power (i.e., 5 mW) is available, a much longer duration of illumination (i.e., several minutes) will be needed. In this sense, the use of a relatively high‐power red laser is desirable. It should be noted that increasing the power of the priming (blue) beam by changing the ratio is not helpful, as this bleaches Dendra2, resulting in lower signal (Dempsey *et al*. 2015; Klementieva *et al*. 2016).

In the original primed conversion experiments with blue and infrared lasers, the authors reported that axial‐confinement was not well achieved without installing a specially‐designed filter (Dempsey *et al*. 2015). Although the exact reason for this difference between their results and our results is unclear, one possible explanation is a chromatic aberration: the blue and infrared lasers might not have been perfectly focused at the same plane in the original report. Because the currently available microscopes (and objective lenses) are not designed to illuminate samples with blue and infrared lasers simultaneously, this kind of aberration may be inevitable. Another (or an additional) possible explanation is the difference in the objective lenses. In this study, the best result was obtained with an objective lens with NA 1.30, which is higher than that of the objective lens used in the original study (NA 1.10; Dempsey *et al*. 2015).

Among the green‐to red photoconvertible fluorescent proteins (PCFPs), Dendra2 was shown to be the best choice for primed conversion (Dempsey *et al*. 2015), leading us to use Dendra2 in the current study. Two recent studies, however, have shown that many other PCFPs can be engineered to become good performers for primed conversion (Mohr *et al*. 2017; Turkowyd *et al*. 2017). The use of these PCFPs would make spatially confined photoconversion experiments even easier in combination with our system.

For the in‐vivo application of spatially primed conversion, we chose to use larval zebrafish as a model organism. By the straightforward application of primed conversion, we have succeeded in highlighting a single cell within a dense, globally fluorescent context. Red Dendra2, produced by primed conversion, was sufficiently bright to reveal the fine morphology of several neuronal types, including motoneurons and several classes of interneurons in the spinal cord. These results show that spatially‐confined primed conversion is a powerful tool for revealing neuronal morphology at a single‐cell resolution.

To express Dendra2 in distinct cell types, we have established Tg[UAS:Dendra2] transgenic fish in this study. Because a large number of Gal4 driver strains are available (Satou *et al*. 2013; Marquart *et al*. 2015; Kawakami *et al*. 2016), Dendra2 can be easily expressed in a variety of cell types simply by crossing Gal4 drivers to the Tg[UAS:Dendra2] fish. Embryos/larvae thus obtained are ready to be used for confined primed conversion. The single‐cell‐resolution photoconversion technique described here can be used not only for revealing cellular morphology but also for monitoring dynamic developmental processes, such as cell migration, morphological changes and divisions.

## Concluding remarks

We have shown that spatially‐confined primed conversion can be achieved in conventional CLSMs without any special modification. CLSMs with blue and red laser lines are very common. In this sense, the method presented here is ready to be used by many researchers, and we believe that the method will become very popular in the future.

## Author contributions

A.T., S.N., and S.H. conceived and designed the study. A.T performed the in‐vitro experiments. Y.K. performed the in‐vivo experiments. I.M. provided a CLSM and helped the in‐vitro experiments. A.T., Y.K., S.N., and S.H. wrote the manuscript.
